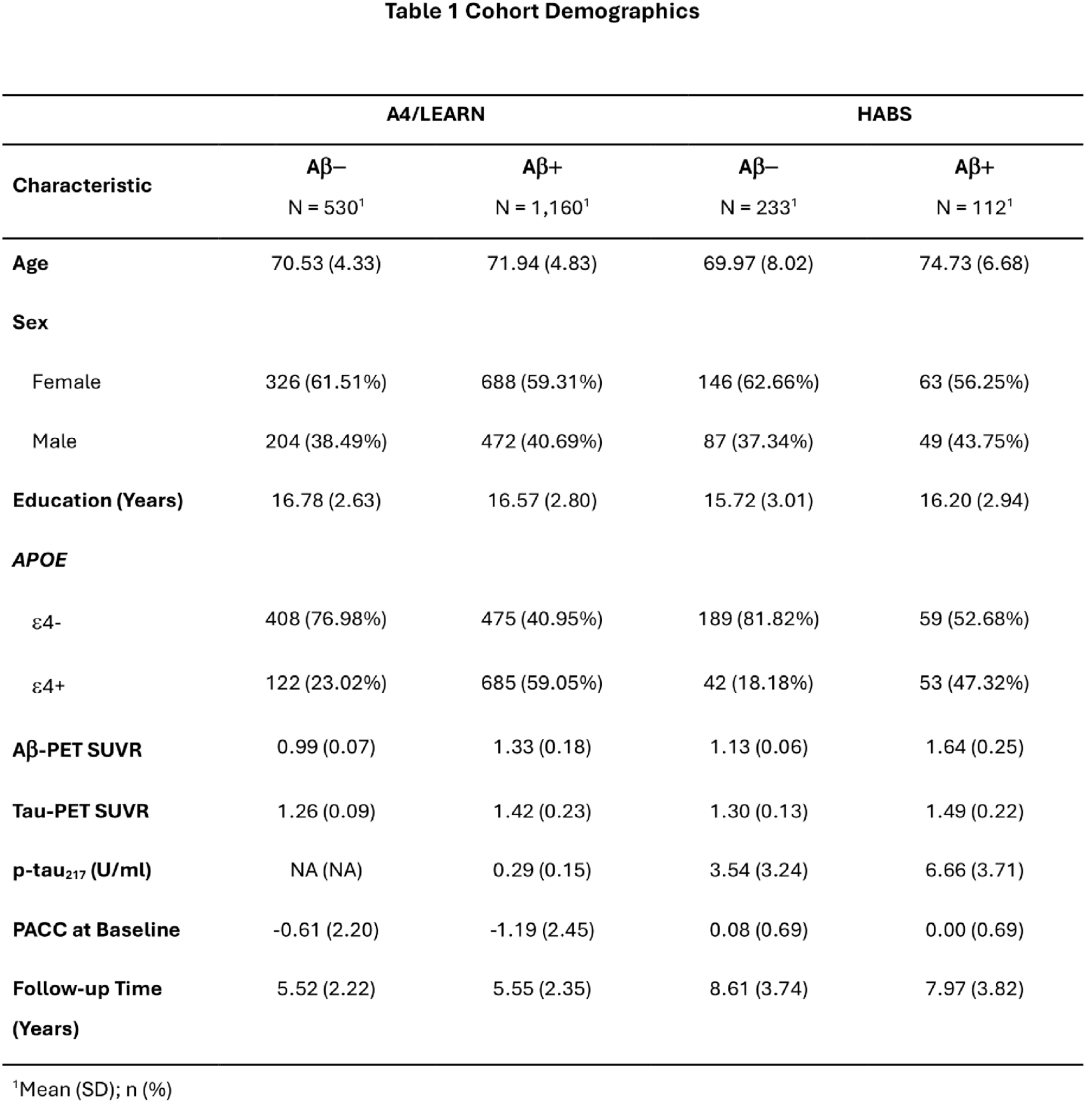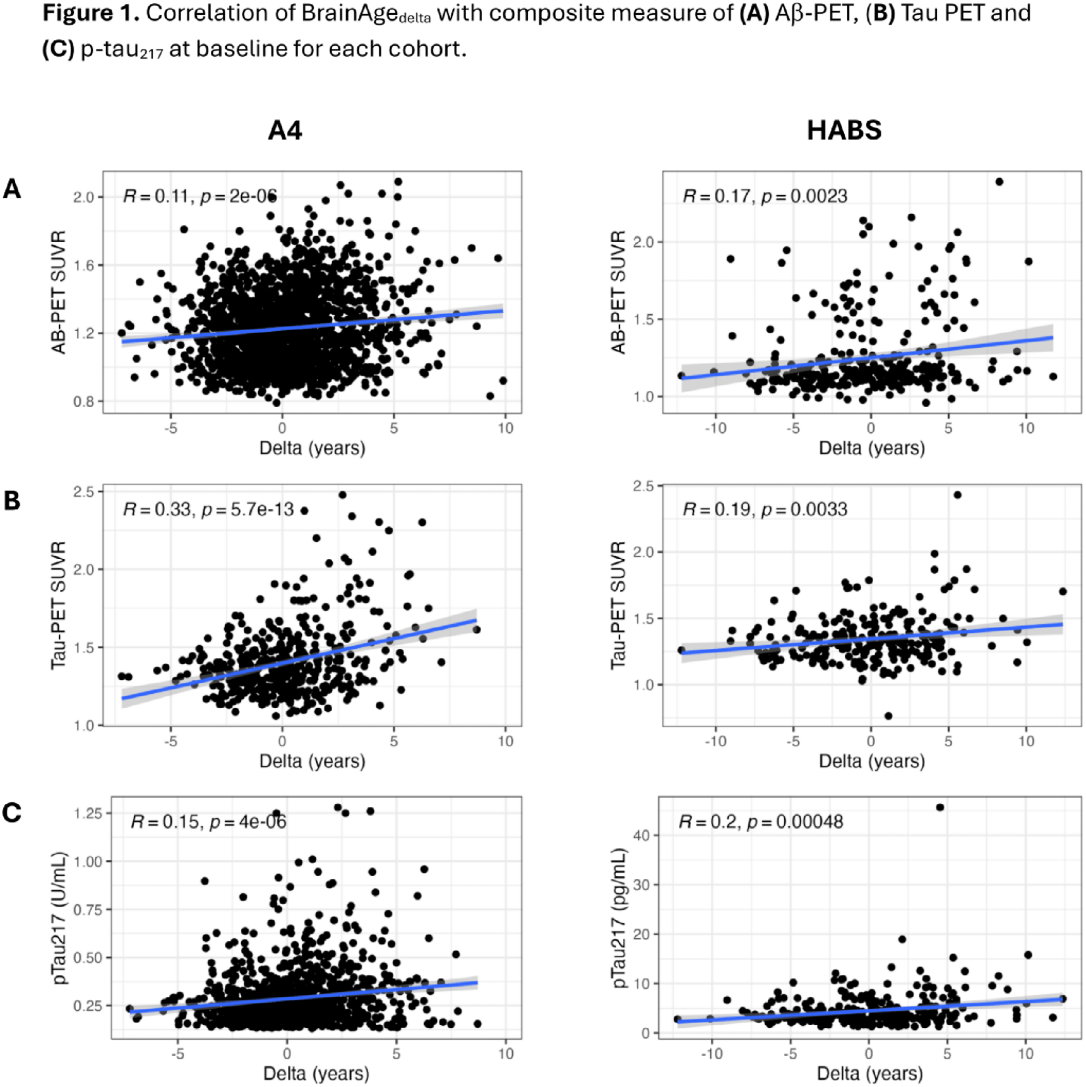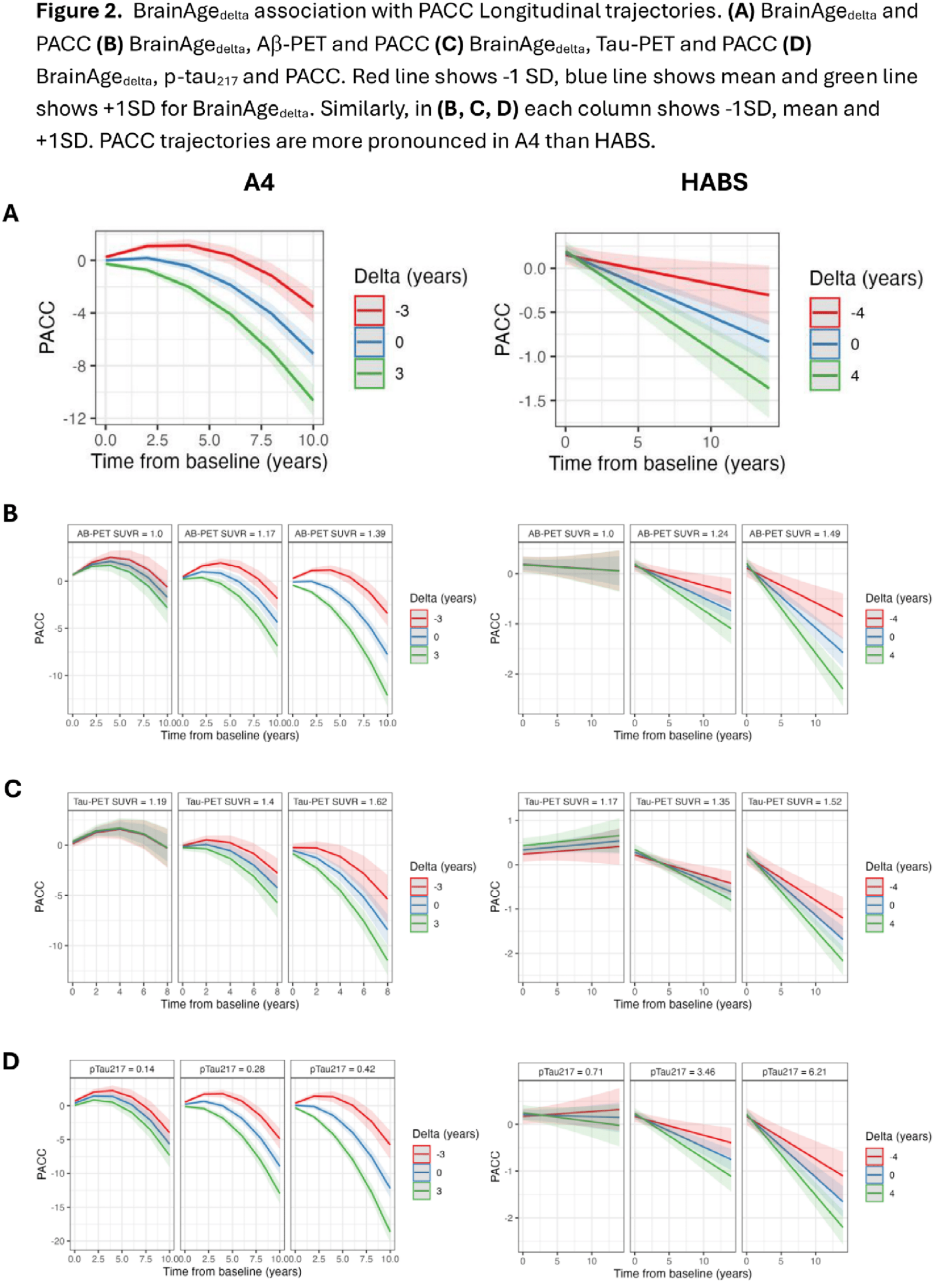# BrainAge moderates associations between AD biomarkers and cognitive decline: findings from A4/LEARN and the Harvard Aging Brain Study

**DOI:** 10.1002/alz70856_106989

**Published:** 2026-01-08

**Authors:** Jorge Garcia Condado, Hannah M Klinger, Colin Birkenbihl, Iñigo Tellaetxe Elorriaga, Mabel Seto, Gillian T Coughlan, Michael J. Properzi, Hyun‐Sik Yang, Asier Erramuzpe, Dorene M. Rentz, Aaron P. Schultz, Jasmeer P. Chhatwal, Keith A. Johnson, Jesus M Cortes, Reisa A. Sperling, Timothy J. Hohman, Michael C. Donohue, Ibai Diez, Rachel F. Buckley

**Affiliations:** ^1^ Massachusetts General Hospital, Harvard Medical School, Boston, MA, USA; ^2^ University of the Basque Country, Leioa, Bizkaia, Spain; ^3^ Biobizkaia HRI, Barakaldo, Bizkaia, Spain; ^4^ Center for Alzheimer's Research and Treatment, Brigham and Women's Hospital, Harvard Medical School, Boston, MA, USA; ^5^ Ikerbasque, the Basque Foundation for Science, Bilbao, Bizkaia, Spain; ^6^ Vanderbilt Memory and Alzheimer's Center, Vanderbilt University School of Medicine, Nashville, TN, USA; ^7^ Alzheimer's Therapeutic Research Institute, University of Southern California, San Diego, CA, USA; ^8^ Melbourne School of Psychological Sciences, University of Melbourne, Melbourne, VIC, Australia

## Abstract

**Background:**

BrainAge models estimate biological brain age based on neuroimaging data, providing a measure of brain health. This metric is particularly relevant in Alzheimer's disease (AD), where accelerated brain aging is exacerbated by β‐amyloid (Aβ) and tau accumulation. We investigated the extent to which BrainAge moderates associations between AD biomarkers and longitudinal cognitive decline across two independent cohorts.

**Methods:**

We examined 1690 participants from A4/LEARN and 349 from HABS (Table 1). Using the Open‐Source tool *AgeML* within each cohort, we built a BrainAge linear regressor model with 5‐fold cross validation using MRI‐T1 volumetric and FreeSurfer cortical thickness ROIs. We compared predicted ages with chronological age to create a BrainAge_delta_. To avoid regressing out sex and *APOEε*4 variance, separate male/female models were built with data from *APOEε*4 non‐carriers and applied to each cohort. We examined BrainAge_delta_ as a moderator of global neocortical Aβ‐PET burden, temporal lobe Tau PET composite and *p*‐tau_217_ associations with longitudinal PACC using linear mixed effects models. We adjusted for random intercepts and slopes, and baseline age, sex, years of education and *APOEε*4. In A4/LEARN we additionally adjusted for cumulative dose and treatment group using a spline model.

**Results:**

Higher levels of Aβ‐PET, Tau‐PET and *p*‐tau_217_ at baseline was significantly correlated with higher BrainAge_delta_ (worse) (Figure 1). BrainAge_delta_ was directly associated with PACC trajectories in both cohorts. It also moderated the association between Aβ and Tau‐PET and PACC trajectories such that higher BrainAge_delta_ was associated with faster cognitive decline with increasing levels of each biomarker. We found the same pattern of effects in *p*‐tau_217_ limited only to the A4/LEARN sample but was trend‐level in HABS (Figure 2).

**Conclusions:**

BrainAge_delta_ is significantly associated with Aβ and tau burden and moderates their association with cognitive decline, supporting previous literature suggesting that BrainAge is a robust marker of brain health. Prioritizing individuals with worse BrainAge for clinical trials could not only effectively reduce screen fails (estimates forthcoming) but is a potentially feasible approach given that it can be calculated from a single T1‐weighted MRI scan. These findings also highlight the importance of age‐independent neurodegeneration patterns to contribute unique signal in models of brain health and pathological progression.